# Influence of handler relationships and experience on health parameters, glucocorticoid responses and behaviour of semi-captive Asian elephants

**DOI:** 10.1093/conphys/coaa116

**Published:** 2021-01-06

**Authors:** J A H Crawley, O Liehrmann, D J Franco dos Santos, J Brown, U K Nyein, H H Aung, W Htut, Z Min Oo, M W Seltmann, J L Webb, M Lahdenperä, V Lummaa

**Affiliations:** 1 Department of Biology, University of Turku, Turku, 20014, Finland; 2 Center for Species Survival, Smithsonian Conservation Biology, Front Royal, VA 22630, USA; 3 Myanma Timber Enterprise, Yangon, 11011 Myanmar; 4 School of Psychology, University of Auckland, 23 Symonds Street, Auckland 1010, New Zealand; 5 Department of Public Health, University of Turku and Turku University Hospital, Turku, 20521, Finland; 6 Centre for Population Health Research, University of Turku and Turku University Hospital, Turku, 20521, Finland

**Keywords:** Animal welfare, glucocorticoids, human–animal interactions, human–animal relationships, mahout, physiology

## Abstract

Declining wild populations combined with accumulating captive populations of e.g. livestock, pets, draught and zoo animals have resulted in some threatened species with substantial proportions of their populations in captivity. The interactions animals have with humans in captivity depend on handler familiarity and relationship quality and can affect animal health, growth and reproduction with consequences for the success of conservation programmes. However, assessments of how specific human–animal relationships affect a range of physiological and behavioural outcomes are rare. Here, we studied semi-captive Asian elephants with detailed records of elephant–handler (mahout) relationships and veterinary management, allowing assessment of multiple welfare indicators in relation to specific mahout–elephant relationship lengths and mahout experience. These included measures of physiological stress (faecal glucocorticoid metabolite [FGM], heterophil:lymphocyte ratio [H:L]), muscle damage (creatine kinase [CK]), immunological health (total white blood cell count [TWBC]) and behaviour (response to mahout verbal commands). We found no evidence that FGM or H:L related to aspects of the mahout–elephant relationship. Longer overall mahout experience (i.e. years of being a mahout) was linked to increased muscle damage and inflammation, but the lengths of specific mahout–elephant relationships were inversely associated with muscle damage in working-age elephants. Elephants responded more to familiar mahouts in behavioural tasks and faster to mahouts they had known for longer. In summary, our results found little evidence that the mahout–elephant relationship affects physiological stress in this population based on FGM and H:L, but mahout experience and relationships were linked to other physiological responses (CK, TWBC), and elephants require behavioural adjustment periods following mahout changes.

## Introduction

Recent human overconsumption and overexploitation of the natural environment have driven drastic population declines across taxa (Dirzo et al., 2014). However, many species also have large populations of individuals in captivity for numerous reasons, such as livestock and pets (globally >1 billion cattle, goats, sheep and dogs and >23 billion poultry; Doherty et al., 2017; FAO, 2020), for laboratory research (79.9–192.1 million animals in 2015; Taylor & Alvarez, 2019), in zoos (>21 000 species; Species360, 2020) and for work (>116 million equids; FAO, 2020). Accumulating captive populations in combination with diminishing wild populations have resulted in some threatened species with a substantial proportion of their total population in captivity. For example, there are six times as many captive tigers as wild (Luo et al., 2008), >20% of Asian elephants and giant pandas live in captivity (Jackson et al., 2019; Kang & Li, 2016) and many species of bird, primate, fish, amphibian and reptile that are common pets face extinction in the wild, such as slow lorises and Spix’s macaw (Tingley et al., 2017).

Human–animal relationships (HARs) have been mostly studied in companion animals and livestock, but also in some laboratory and zoo animals (Hosey & Melfi, 2014b). HARs have been found to significantly affect animal welfare, and effects depend on the species, the type and quality of the HAR and the familiarity of the human. A lot of research has investigated animals’ general fear of humans (Hosey, 2008), finding that it influences their physiological stress, growth, health and reproduction (Hemsworth, 2003). Studies have also assessed HARs in relation to animals’ affinity to specific people that can be distinct from their general fear of humans (Carlstead, 2009), especially in relation to managed animals’ interactions with specific caretakers (Hosey, 2008). For example, zoo-managed clouded leopards that spent more time with fewer, but familiar keepers had lower faecal glucocorticoid metabolite (FGM) concentrations than those that were exposed to many different keepers (Wielebnowski et al., 2002), and for multiple felid species, those with more interactive keepers had overall greater reproductive success (Mellen, 1991). Primates experiencing positive caretaker interactions showed more grooming and playing behaviours and reduced abnormal behaviours (Baker, 2004), but effects appear to be context dependent; caretaker presence negatively affected laboratory primates, and zoo visitors often elicit aggression in primates (Hosey & Melfi, 2014b).

Managed animals experience varying degrees of captivity, and those with partial freedom are often termed ‘semi-captive’. Semi-captive animals usually range, forage and socialize in their natural environment but are also influenced by humans; for example, receiving veterinary care (Franco dos Santos et al., 2020), food supplementation (Dellatore et al., 2014) and managed enclosures (Standley et al., 2011). Semi-captivity lacks many of the concerns often associated with full captivity, such as restricted movement, absence of social structure or opportunities or loss of survival skills. Thus, semi-captive animals can play a role in *in situ* conservation or reintroduction efforts, being more adapted to their natural habitat than fully captive individuals (Keulartz, 2015). Their life history traits are also sometimes used as a proxy for wild individuals as they experience more natural ecological conditions than fully captive individuals (Clubb et al., 2008). While it is important to understand the impacts of HARs across different captive contexts, few studies have focused on semi-captive animals (Hosey & Melfi, 2014b). Tourists have been shown to have negative effects on semi-captive orangutans (Dellatore et al., 2014), although results are mixed for Asian elephants (Brown et al., 2020; Kontogeorgopoulos, 2009). These relationships are more comparable to visitor effects on zoo animals, which can be stressful or benign (Davey, 2007), than more specific caretaker interactions that tend to be positive in zoos but are little understood in a semi-captive context (Carlstead et al., 2019; Hosey, 2008).

HARs are particularly important for Asian elephants as 24–29% of them are currently managed by humans (Jackson et al., 2019). The importance of these relationships has been clearly demonstrated in studies of zoo elephants, with elephants whose keepers considered their bonds to be stronger having lower serum cortisol levels (Carlstead et al., 2019). Furthermore, elephants in the tourism industry whose management involved more interactions with their handlers and visitors showed lower FGMs, although the opposite has also been found (Brown et al., 2020). Over 90% of captive Asian elephants (~15 000) are managed in free contact (humans and elephants share the same space) environments in Asia, by one or more traditional handlers (mahouts) who are almost entirely responsible for their care (Sukumar, 2003), yet we know very little about the mahout–elephant relationship. Changes have occurred recently within the mahout profession across Asia, with mahouts tending to be younger and less experienced (Crawley et al., 2019), having fewer employment options and exhibiting higher job turnover than in the past (Srinivasaiah et al., 2014). Elephant management practises continue to evolve, especially related to elephant tourism (Bansiddhi et al., 2018), but it is unclear how these changes are influencing elephant well-being.

An extensive set of studies of elephants in Thailand and North America have provided insight into factors impacting elephant health and welfare, finding that environmental conditions, social dynamics, exposure to tourists/visitors, diet and exercise opportunities can influence physical and physiological function, including FGMs (Brown et al., 2020). However, few studies have assessed specific mahout–elephant relationships, flagged as important for future studies (Brown et al., 2020). One could assume that replacing long-term, experienced mahouts with frequently changing, less experienced mahouts would negatively affect elephants; however alternatively, long-time caretakers could become complacent over time and pay less attention to their animals, suggested by studies on zoo animals in the USA (Carlstead, 2009) and semi-captive elephants in India (Srinivasaiah et al., 2014). Epidemiological and physiological monitoring can provide insights into animal health and welfare both within and across populations, including elephants (Brown et al., 2020), although most studies have been conducted on more intensively managed animals that are more accessible.

This study investigated HARs in the world’s largest semi-captive population of Asian elephants—the timber elephants of Myanmar. These elephants have logbooks recording mahout changes through time, which we have combined with information from interviews with 190 mahouts and 18 head mahouts to calculate specific relationship lengths, mahout experience and mahout age. The elephants’ training allowed us to obtain faecal and blood samples and to conduct behavioural assessments to investigate a range of welfare indicators in response to specific mahout–elephant relationship variables. To assess physiological stress, we measured FGM concentrations, which increase in response to a variety of stressors (Brown et al., 2019) and the ratio of heterophil to lymphocyte white blood cells (H:L ratio), which increases in response to stress or infection (Davis et al., 2008). We also measured creatine kinase (CK), an indicator of physical stress that increases with muscle damage (Chulayo & Muchenje, 2013), and immunological health through analysis of total white blood cell counts (TWBC) that react to inflammation or infection (Fowler & Mikota, 2006). Finally, we measured elephant behavioural responses to commands given by familiar and unfamiliar mahouts to determine compliance and response time. We hypothesized that stronger mahout relationships would be associated with lower stress measures and more cooperative behaviours in their elephants**.**

## Methods

### Study Population

We study elephants owned by the Myanma Timber Enterprise (MTE), who manage ~3000 elephants distributed across the country, with the largest populations in the Sagaing (~1000), Bago (~900) and Kachin (~900) regions of Myanmar (Hedges et al., 2018). Elephants work ~5–8 hours a day depending on their age and size and the season, but rest during the hot season (March–May). They are considered semi-captive as they are released each evening (front legs sometimes fettered) to range, forage, socialize and mate in their natural habitat. This mostly entails socializing with elephants within their working group, but they can also encounter other MTE elephants in adjoining areas, as well as wild elephants. Each elephant is paired with a mahout at the age of 4 years after being tamed, and kept in a work group of around six elephants managed by a head mahout (sin-gaung). Each region of ~100 elephants is overseen by a senior head mahout (sin-oke) and a veterinarian. Each mahout collects his elephant from the forest every morning and is responsible for its daily care, such as bathing, and monitoring health, diet, defecation and sleeping habits. Each elephant has a logbook that includes information on birth date, offspring, sex, origin (captive born/wild caught), veterinary interventions and mahout information, recorded monthly by the local MTE veterinarian.

### Data Collection

Faecal samples were collected roughly monthly from a total of 151 elephants in the Sagaing region of Myanmar between February 2012–April 2018 spanning all months of the year, and blood samples were collected from a total of 148 elephants between November 2015–April 2018, in March/April, July and November, corresponding to the beginning of each season (hot: March–May, monsoon: June–Oct, cold: Nov–Feb; see Table 1 for sample sizes). Behavioural tests were conducted and filmed in March–April 2017 and 2018 and behaviours assessed in early 2019 by a single observer using BORIS (Friard & Gamba 2016). Data and samples were collected according to the University of Turku’s ethical rules.

**Table 1:** Summary of data included in models A–F, for (i) elephant relationship with their mahout and (ii) mahout total experience in A–D, and (i) mahout identity and (ii) mahout relationship length in E–F

**Variable**	**Model**	**No. Obs** ^**a**^	**No. Inds** ^**b**^	**M**^**c**^**-E**^**d**^ **relationship** **(years) Cont**^**e**^		**M Total experience (years) Cont**		**M Age (years) Cont**		**E Age (years) Cont**		**E Sex (No. Inds) Cat** ^**f**^		**Season (No. Inds) Cat**			**M Identity (No. Obs) Cat**		**Command Rate (/sec) Cont**		**Calling M Total experience (No. Obs) Cat**			
				Range	Mean	Range	Mean	Range	Mean	Range	Mean	F^**g**^	M^**h**^	Hot	Cold	Wet	Own	Other	Range	Mean	1	2	3	4
**(A) FGM**	(i)	1964	151	0.0–12.0	1.5	-	-	-	-	4.1–71.3	16.5	89	62	596	705	663	-	-	-	-	-	-	-	-
	(ii)	1402	138	-	-	0.0–24.0	4.0	11.0–58.0	24.0	4.2–71.3	17.0	82	56	438	522	442	-	-	-	-	-	-	-	-
**(B) H:L Ratio**	(i)	370	148	0.0–12.0	1.7	-	-	-	-	4.2–71.3	21.3	89	59	195	100	75	-	-	-	-	-	-	-	-
	(ii)	341	138	-	-	0.0–29.0	4.2	14.0–59.0	25.0	4.2–71.3	21.9	83	55	184	89	68	-	-	-	-	-	-	-	-
**(C) CK**	(i)	329	122	0.0–12.0	1.7	-	-	-	-	4.4–71.3	20.4	73	49	152	102	75	-	-	-	-	-	-	-	-
	(ii)	307	116	-	-	0.0–29.0	4.1	14.0–59.0	25.2	4.4–71.3	21.2	68	48	148	91	68	-	-	-	-	-	-	-	-
**(D) TWBC**	(i)	397	148	0.0–12.0	1.7	-	-	-	-	4.2–71.3	20.8	89	59	220	102	75	-	-	-	-	-	-	-	-
	(ii)	367	138	-	-	0.0–29.0	42	14.0–59.0	25.0	4.2–71.3	21.4	83	55	208	91	68	-	-	-	-	-	-	-	-
**(E) Task success**	(i)	136	81	-	-	-	-	-	-	5.7–71.4	23.6	73	63	136	0	0	72	64	0.0–2.2	0.9	29	31	35	41
	(ii)	135	80	0.0–11.0	0.9	-	-	-	-	5.7–71.4	23.7	72	63	135	0	0	-	-	0.0–2.2	0.9	29	35	31	40
**(F) Response time**	(i)	88	66	-	-	-	-	-	-	6.2–71.4	26.7	46	42	88	0	0	55	33	0.0–1.9	0.9	20	24	20	24
	(ii)	87	65	0.0–7.0	0.9	-	-	-	-	6.2–71.4	26.9	45	42	87	0	0	-	-	0.0–19	0.9	20	24	20	23

a ^a^observations, ^b^individuals, ^c^mahout, ^d^elephant, ^e^continuous, ^f^categorical, ^g^female, ^h^male

Mahout identities and the dates the mahouts were paired with their elephants were collected in three different ways: from interviews with 190 mahouts in March–April 2017 and 2018 (see Crawley et al. 2019), with 18 head mahouts in March–April 2018, and from 53 elephant logbooks. From this information, we calculated the length of time each mahout had been paired with his elephant at each measurement date. We also recorded a mahout’s total time working with elephants and their age on the date of measurement from mahout interviews, but this information was not available through the other two methods.

### Faecal samples: Faecal Extraction and FGM Analysis

Faecal samples were collected in the morning soon after defecation to reduce diurnal variation, frozen at −20°C until dried at 50°C for 24 hours and analysed at the Veterinary Diagnostic Laboratory in Chiang Mai, Thailand. Samples (0.1 g) in 5 ml of 90% ethanol were extracted twice by boiling in a water bath for 20 minutes and adding 100% ethanol as needed to maintain volume. Samples were centrifuged and the combined supernatants dried under air in a 50°C water bath. Samples were reconstituted by vortexing for 1 minute in 3 ml of ethanol, drying again, and finally resuspended in 1 ml of methanol. Extracts were diluted 1:3 in assay buffer and stored at −20°C until analysis.

Concentrations of FGM were determined using a double-antibody enzyme immunoassay (EIA) validated for Asian elephants that relied on a polyclonal rabbit anti-corticosterone antibody (CJM006) as described by Watson et al. (2013) and Norkaew et al. (2018). Second antibody-coated plates were prepared by adding 150 μl of anti-rabbit IgG (0.01 mg/ml) to each well of a 96-well microtiter plate, and incubating at room temperature for 15–24 hours. The wells were then emptied and blotted dry, followed by adding 250 μl of blockingsolution and incubating for 15–24 hours at room temperature. After incubation, wells were emptied, blotted and dried in a Sanpla Dry Keeper (Sanplatec Corp., Auto A-3, Japan) with loose desiccant in the bottom. Dried plates were heat sealed in a foil bag with a 1 g desiccant packet, and stored at 4°C until use.

**Figure 1:**
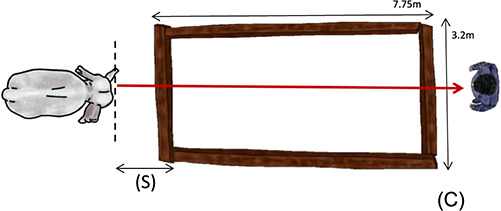
Behavioural test set-up, showing the mahout calling to the elephant across the arena. (S) shows the period between the first command given to the first step into the arena during which response time and command rate are calculated. The red arrow is the trajectory the elephant has to walk through to complete the test for task success. (C) shows the position of the camera

Samples or corticosterone standards (50 μl) followed immediately by corticosterone-horseradish peroxidase (25 μl) were added to each well except for non-specific binding wells, followed by 25 μl of anti-corticosterone antibody, and incubated at room temperature for 1 hour. Plates were washed four times with buffer (1:20 dilution, 20X Wash Buffer Part No. X007; Arbor Assays, MI) and 100 μl of tetramethylbenzidine substrate solution was added, followed by incubation for 45–60 minutes at room temperature without shaking. Absorbance was measured at 405 nm. The intra-assay variation was <10% as all samples with duplicate intra-assay coefficients of variability >10% were reanalysed. The inter-assay variation was 10.28% and 6.78% for high- and low-quality control samples, respectively. Assay sensitivity (minimum detection limit) was 0.099 ng/g faeces. Prior to analysis, we removed outlier values of <10 ng/g(3 observations).

### Blood samples: Total White Blood Cell Count, Heterophil:Lymphocyte Ratio, Creatine Kinase

Blood samples were collected in the morning from an ear vein into a vacuette® (Greiner Bio-One, Austria) with either ethylenediaminetetraacetic acid (for TWBC/H:L Ratio) or serum separator/clot activator (for CK) and refrigerated for <24 hours before processing. We used a microscope at ×10 magnification to count white blood cells in a Neubauer haemacytometer, after lysing red blood cells with Türk’s solution. We stained blood smears using a Romanowsky stain and classified 100 cells at ×40 magnification as heterophils, lymphocytes, monocytes, basophils or eosinophils, and then calculated a heterophil to lymphocyte ratio for each sample. For CK, vacuettes were spun in a centrifuge at 3400 rpm for 20 minutes to obtain 2 ml of serum, which was frozen at −20°C until analysis at the Crown Laboratory, Yangon, Myanmar, using an IDEXX VetTest® analyser (IDEXX, USA).

### Behavioural tests

Behavioural tests assessed elephant responses to mahout commands. An arena (7.75 m × 3.2 m) was marked using wooden planks on the floor (Fig. 1) and each mahout stood at the far end and gave verbal commands for the elephant to cross the arena towards him. This was repeated both with the elephant’s own mahout, and the mahout of another elephant, with the order randomized. We recorded three main measures: (i) task success (a binomial measure of an elephant’s success at crossing the arena), (ii) response time (duration from the first command given until the elephant’s first step into the arena; [S] in Fig. 1) and (iii) command rate (the number of commands given per second in period [S]). Each leg of the task was only performed once per occasion with each elephant to avoid habituation to the experimental design.

## Statistical Analysis

### Physiology

We fit four sets of models using R (version 3.5.3; R Core Team, 2019), to assess how the mahout–elephant relationship affects: (A) FGM concentration, (B) H:L ratio, (C) CK and (D) TWBC, as response variables. Linear mixed-effects models were fit using the lmer function (Bates et al., 2015) for models (A), (B) and (D) with normal distributions, after log-transformation of the response variables in (A) and (B)—after adding a constant of 1 to avoid negative values for (B). This and following transformations refer to natural logarithms. Models (C) were fit using the glmmTMB function (Brooks et al., 2017) with a negative binomial distribution accounting for zero inflation. We assessed whether these measures correlated with the elephant’s (i) relationship length with their mahout and (ii) their mahout’s total experience and age (see Table 1 for variable descriptors). Questions (i) and (ii) were assessed in separate models as information for (ii) was only available for a subset of elephants whose mahout was interviewed. Models (ii) included the potentially correlated traits of mahout age and experience, but the correlation coefficient never exceeded 0.5. In each model, we tested whether log-transforming the relationship length and total experience (after adding a constant of 1) terms, improved model predictive performance, as we expected effects to be strongest shortly after mahout change, and to lessen over time. We also tested quadratic effects of elephant age where exploratory plots suggested this was appropriate. Each model included elephant age, sex and collection season as fixed effects, and a random intercept of ID to account for pseudo-replication of repeated individuals (see Table 1 for variable breakdown). We included a random intercept of measurement batch (eight levels) in FGM models, to account for temporal measurement differences, and a fixed effect of days between collection and analysis (6–221 days, mean = 91) in the CK models to account for storage time effects. We scaled continuous predictors to aid model convergence and mean-centred age and experience variables for which comparisons to zero were not meaningful. We tested for two-way interactions between mahout relationship length or total experience and elephant age/sex in case effects were age or sex dependent. We compared model predictive performance using the Akaike Information Criterion and used the ANOVA function (R Core Team, 2019) to determine test statistics. When drawing conclusions, we retained non-significant biologically important terms, although results were consistent when removing non-significant terms before comparisons. We also refit all models on datasets of only elephants <20 years old for which we had most observations, and our main results were consistent. We did not include birth origin (captive born/wild caught) as a term in models as over 75% of our sample were captive-born, with an average time since capture for wild-caught elephants of 40 years and because previous research has found that detrimental effects of capture are mostly realized within the first decade post-capture (Lahdenperä et al., 2018). Predicted effects were calculated using ggpredict (Lüdecke, 2018), with mean continuous variables and categorical reference levels stated in the results.

### Behaviour

To analyse the behavioural data, we fit two generalized linear models using the brm function (Bürkner, 2017) with a binomial response variable of (E) task success (1: success/0: fail) using a Bayesian framework as the data had underlying structure (zeros biased towards other mahout and young elephants) limiting REML model convergence. We used informative priors drawn from a Cauchy distribution for continuous predictors (mean = 0, SD = 2.5). We examined effect sizes of regression coefficients and judged their importance based on whether credible intervals encompassed zero. Models assessed whether mahout familiarity affected task success, in terms of (i) mahout status (own/other mahout), and (ii) relationship length between the elephant and calling mahout (0 for other mahouts). We included fixed effects of elephant age, sex and the calling mahout’s command rate (see Table 1) and tested two-way interactions between mahout status or relationship length and elephant age and sex. We also tested inclusion of the calling mahout’s total experience as a fixed effect to account for differences in expertise, categorized into quartiles (‘1’: <24 months, ‘2’: 24–38 months, ‘3’: 39–119 months, ‘4’: >120 months), but this did not improve model predictive performance. We included a random effect of individual ID to account for pseudo-replication. Although there were few repeats per individual, results were consistent in a model excluding this random term, and the Bayesian approach accounts for uncertainty in random effects. We selected models using k-fold cross validation (k = 10), choosing the simplest model with the lowest Kfold-IC (Vehtari et al., 2019).

We next assessed elephants’ (F) response times in relation to the same mahout familiarity measures for only successful tasks (see Table 1). We fit these models using the glmmTMB function with a Poisson distribution accounting for zero inflation. We tested and accounted for the same variables as model E, but the ID random effect was removed as there were fewer repeated observations and it accounted for little variance. We compared models and assessed model predictive performance as described for models A–D.

## Results

**(A) Faecal Glucocorticoid Metabolite Concentrations**

**Figure 2:**
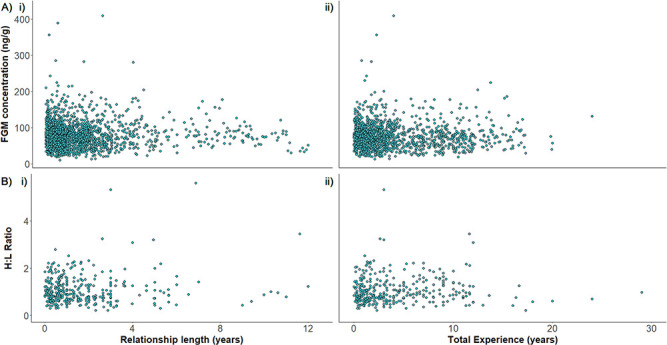
Elephant stress response represented by (A) faecal glucocorticoid metabolite and (B) heterophil:lymphocyte ratio in relation to (i) their relationship length with their mahout and (ii) their mahout’s total experience

FGM concentrations of elephants ranged from 10.2–409.7 ng/g (mean [±SE] =78.6 ± 0.8 ng/g) but did not relate to their relationship length with their mahout (i: χ^2^_1_ = 0.39, *P* = 0.534), their mahout’s total experience (ii: χ^2^_1_ = 0.004, *P* = 0.951) or their mahout’s age (ii: χ^2^_1_ = 0.56, *P* = 0.455); see Fig. 2Ai and Aii**,** Tables S1i and S1ii. FGM concentration did not correlate with an elephant’s sex (i: χ^2^_1_ = 0.09, *P* = 0.762; ii: χ^2^_1_ = 0.08, *P* = 0.784) or age (i: χ^2^_1_ = 0.41, *P* = 0.523; ii: χ^2^_1_ = 0.50, *P* = 0.481), but it significantly differed by season (i: χ^2^_2_ = 10.94, *P* < 0.01; ii: χ^2^_2_ = 7.70, *P* < 0.05), being the highest with a mean of 80.7 ± 1.31 ng/g in the cold season compared to 77.7 ± 1.27 ng/g and 77.1 ± 1.60 ng/g in the monsoon and hot seasons, respectively.

**(B) Heterophil:Lymphocyte ratio**

The elephants’ H:L ratios ranged from 0.2–5.6 (mean [±SE] = 1.07 ± 0.03) and did not correlate with the elephants’ relationship length with their mahout (i: χ^2^_1_ = 1.87, *P* = 0.172), their mahout’s total experience length (ii: χ^2^_1_ = 0.01, *P* = 0.917), or their mahout’s age (ii: χ^2^_1_ = 0.14, *P* = 0.706); see Fig. 2Bi and Bii, Tables S2i and S2ii. An elephant’s H:L ratio did not significantly relate to their age (i: χ^2^_1_ = 1.45, *P* = 0.229; ii: χ^2^_1_ = 1.06, *P* = 0.303), or sex (i: χ^2^_1_ = 0.10, *P* = 0.747; ii: χ^2^_1_ = 0.003, *P* = 0.955), but significantly differed between seasons (i: χ^2^_2_ = 15.93, *P* < 0.001; ii: χ^2^_2_ = 12.88, *P* < 0.01), the highest in the cold season with a mean (±SE) of 1.25 (±0.07), compared to 0.94 (±0.05) and 1.03 (±0.04) in the monsoon and hot seasons, respectively.

**(C) Creatine Kinase**

**Figure 3:**
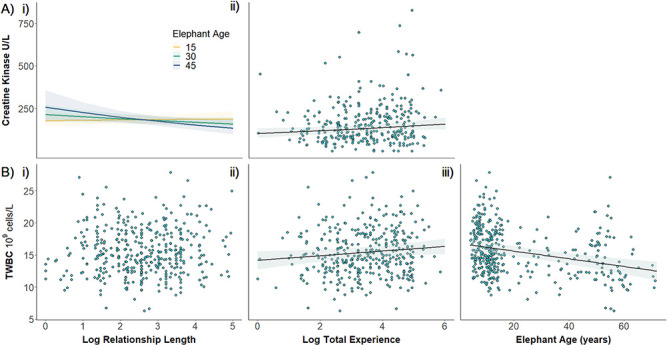
Elephant (A) muscle damage (ck) and (B) immunological response (TWBC) in relation to their (i) log relationship length with their mahout, (ii) mahout's log total experience length (both originally in months) and (iii) age. Points show the raw data, and lines show predicted levels. Shaded areas show the 95% confidence intervals

Elephants’ CK ranged from 0–831 enzyme units/litre (U/L) (mean [±SE] =159 ± 7 U/L), and there was a significant negative interaction between logarithmic relationship length and elephant age (i: χ^2^_1_ = 5.64, *P* < 0.05; Fig. 3Ai; Table S3i). The correlation between log relationship length and CK was slightly positive for young elephants, but CK declined with longer relationships from age 18 onwards. For example, the CK of a 15-year-old elephant with a 3-month relationship with its mahout was similar to that after a 4.5 year-long relationship, predicted to increase from 180 U/L to 185 U/L (for females in the hot season after 95 days storage), whereas the CK of a 30-year-old elephant was predicted to decrease from 201 U/L to 168 U/L, and the CK of a 45-year-old elephant from 225 to 152 U/L at the same relationship lengths. An elephant’s CK was positively correlated to their mahout’s logarithmic total experience (ii: χ^2^_1_ = 6.72, *P* < 0.01), with a predicted CK value of 143 U/L if their mahout had a total of 3 months of experience, compared to 207 U/L with 12 years of experience (Fig. 3Aii; Table S3ii). The negative interaction between elephant age and total mahout experience did not reach significance (ii: χ^2^_1_ = 2.20, *P* = 0.139), and neither did an additive elephant age term in model ii) (ii: χ^2^_1_ = 0.01, *P* = 0.904). Mahout age did not significantly affect CK (ii: χ^2^_1_ = 2.51, *P* = 0.113), nor did elephant sex (i: χ^2^_1_ = 1.37, *P* = 0.242; ii: χ^2^_1_ = 0.38, *P* = 0.536) in either model, consistent with Franco dos Santos et al. (2020). CK differed by season (i: χ^2^_2_ = 23.81, *P* < 0.001; ii: χ^2^_2_ = 20.55, *P* < 0.001), the highest in the hot season, with a mean (±SE) of 192.7 U/L (±8.4) compared to 124.7 (±14.1) and 133.5 (±12.7) U/L in the monsoon and cold seasons, respectively. CK was also significantly negatively correlated with storage time in both models (i: χ^2^_1_ = 60.66, *P* < 0.001; ii: χ^2^_1_ = 56.35, *P* < 0.001).

**(D) Total White Blood Cell Count**

TWBC counts ranged from 6.3–27.9 × 10^9^ cells/L (mean [±SE] = 15.5 ± 0.2 x 10^9^/L) and did not depend on an elephant’s logarithmic relationship length with their mahout (i: χ^2^_1_ = 0.53, *P* = 0.465; Fig. 3Bi), or their mahout’s age (ii: χ^2^_1_ = 0.05, *P* = 0.821; Tables S4i & S4ii). TWBC counts increased slightly with mahout total experience, though this did not reach significance (ii: χ^2^_1_ = 3.25, *P* = 0.072). When removing the mahout age term, however (which did not improve model predictive performance and was not deemed biologically significant), this reached significance (ii: χ^2^_1_ = 4.21, *P* = 0.040; Fig. 3Bii). The predicted TWBC count of an elephant with a mahout of 3 months total experience was 14.5 × 10^9^/L compared to 16 × 10^9^/L for an elephant with a mahout of 12 years’ experience, increasing by >10% (predictions for females in the cold season). TWBC counts significantly decreased with elephant age (i: χ^2^_1_ = 18.08, *P* < 0.001; ii: χ^2^_1_ = 17.33, *P* < 0.001; Fig. 3Biii) but did not depend on elephant sex, consistent with Franco dos Santos et al. (2020) (i: χ^2^_1_ = 0.87, *P* = 0.350; ii: χ^2^_1_ = 0.39, *P* = 0.534), or measurement season (i: χ^2^_2_ = 1.95, *P* = 0.378; ii: χ^2^_2_ = 1.95, *P* = 0.378) in this sample.

**(E) Task Success**

**Figure 4:**
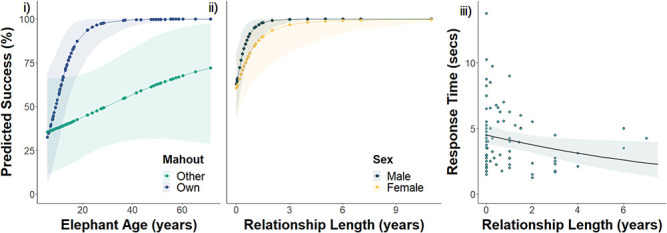
Elephant behavioural responses, with (i–ii) showing predicted task success in relation to (i) the calling mahout’s identity, depending on their age, and (ii) their relationship length with the calling mahout, depending on their sex and (iii) showing elephant response time depending on their relationship length with the calling mahout. Lines show predicted values, with (i) and (ii) based on the interactive models in Tables S5i and S5ii, respectively, and (iii) on the model shown in Table S6ii (for females in (i) and (iii)). Points in (iii) show raw response times, and shaded areas show 95% confidence intervals

The majority of elephants (79%) completed the task when their own mahout was calling, compared to 51% with another mahout. Statistically, elephants were more successful when their own mahout was calling, but this depended on the elephant’s age (lower 95% CI = 0.06, upper 95% CI = 0.47; Fig. 4i; Table S5i). Older elephants responded more to their own mahout than younger elephants, and although also true when responding to another mahout, the effect was less pronounced. For example, a 14-year-old female elephant had an 81% predicted probability of success with its own mahout and a 37-year-old had a predicted success of >99% with its own mahout, whereas with another mahout calling the probability was only 47% and 56% at the same ages. Task success however did not depend on elephant sex, mahout total experience or command rate. Model (ii) assessing the effect of mahout relationship length found a similar age dependence, though less strong (lower 95% CI = 0.00, upper 95% CI = 0.03; Table S5ii), and the effect of relationship length on an elephant’s success depended more on elephant sex (lower 95% CI = 0.02, upper 95% CI = 0.21). Males were more successful and less dependent on relationship length (Fig. 4ii): a male with a year-long relationship with the calling mahout had 95% predicted success and with a3-year-long relationship 99%, whereas females only had 86% and 97% predicted successes with the same relationship lengths.

**(F) Response Time**

Although elephants responded faster on average to their own mahouts (mean = 4.2 ± 0.29 sec), than other mahouts (mean = 4.4 ± 0.5 sec), these were not significantly different (i: χ^2^_1_ = 1.87, *P* = 0.172; Table S6i), and response time did not depend on elephant age (i: χ^2^_1_ = 2.81, *P* = 0.09; ii: χ^2^_1_ = 2.72, *P* = 0.099) or sex (i: χ^2^_1_ = 0.09, *P* = 0.765; ii: χ^2^_1_ = 0.01, *P* = 0.941). Elephants responded faster to mahouts calling more frequently (i: χ^2^_1_ = 11.25, *P* < 0.001; ii: χ^2^_1_ = 10.34, *P* < 0.01), and those they had known for longer (ii: χ^2^_1_ = 5.30, *P* < 0.05; Fig. 4iii; Table S6ii); female elephants with a 7.5-year relationship with the calling mahout were predicted to respond in 2.6 seconds, compared to 4.4 seconds with no prior relationship.

## Discussion

Here we show for the first time that mahout relationships can affect the physiology and behaviour of elephants in a semi-captive setting. Although we found no evidence that the length of the mahout–elephant relationship or how long mahouts had been working with elephants in total affected adrenal glucocorticoid activity or heterophil: lymphocyte ratios**,** mahout relationships and experience were linked to other physiological responses indicating muscle damage and inflammation. In addition, elephants appear to require behavioural adjustment periods following mahout changes as indicated by elephants responding more to familiar mahouts and faster to those they had known for longer. This has important implications seeing as long-term mahout–elephant relationships are becoming less common.

We found no evidence that FGM concentrations or the H:L ratio as indicators of physiological stress were related to the length of the mahout-elephant relationship or the mahout’s total years of experience. This may suggest the mahout relationship is not a primary factor driving physiological stress responses in this population, or that any negative effects of inexperienced mahouts are buffered by other factors. A past study also found that mahout experience was not correlated with working elephant condition or welfare indicators (Chatkupt et al., 1999), but rather factors such as location, work activities and shade/food availability were more important. A study of semi-captive elephants in India also suggested that potentially stressful effects of working may be buffered by the benefits of elephants living in a natural environment (Kumar et al., 2019). Another found adequate diet, exercise, rest areas and social opportunities to be most important for elephant welfare (Brown et al., 2020). Diet and rest areas are not great concerns for MTE elephants as they forage naturally and roam the forests at night. Moreover, group demographics, social opportunities and work schedules are regulated by management. Studies often consider interactions in terms of time spent together or engagement in a structured activity (Hosey & Melfi, 2014a)—factors that are again reasonably consistent for MTE elephants, as daily routines are regulated by management. Although we cannot be certain that declining relationship lengths do not pose a threat to elephant well-being, it does not appear to affect the physiological stress indicators we measured (i.e. FGM or H:L). Finally, Burmese mahouts are considered particularly skilled and knowledgeable (Sukumar, 2003), and the extensive handling system, regular veterinary visits and management protocols may maintain quality care despite individual mahout changes. Furthermore, the fact that short relationships are becoming the norm may explain why we did not see any evidence of mahout relationship variables correlating with these indicators of physiological stress as we have few long-term relationships to compare to.

The mahout–elephant relationship influenced an elephant’s CK levels, which indicate muscle damage and strain (Chulayo & Muchenje, 2013), but the effect depended on elephant age. There was little effect in younger elephants, though CK slightly increased with longer relationships, but as elephants aged, CK decreased logarithmically with longer relationships. The weak trend in juveniles may reflect younger elephants unable to work when first paired with a new mahout, but gradually able to do more as relationships lengthen. There was a more defined, negative correlation between relationship length and CK from age 18 onwards, which corresponds with elephants entering the workforce and could reflect new mahouts using more physical persuasion while establishing a working relationship but gaining trust and understanding over time, learning to interpret the elephant’s individual behaviours. It is important to note that although only older elephants can have relationship lengths over a certain threshold, many older elephants also have short relationships, and a logarithmic relationship length term should account for this bias to an extent.

In contrast to data related to length of specific mahout–elephant relationships, CK and to some extent TWBC count logarithmically increased with the mahout’s total experience. One explanation could be that logging work requires skill and expertise, and only experienced mahouts perform the toughest operations, most likely to cause muscle strain and inflammation, thus increasing CK and TWBC count. Alternatively, more experienced zoo keepers judged their animals as more fearful of humans than less experienced keepers, possibly due to keeper complacency over time, or gained knowledge of the fear response (Carlstead, 2009), so perhaps mahouts may become complacent over time and pay less attention to their elephants. Elephants in India were less fearful and aggressive and more social towards their assistant mahout than towards their main, more experienced mahout, although this could be due to spending less time with main mahouts (Srinivasaiah et al., 2014), or perhaps main mahouts are more dominant. Finally, views surrounding animal handling and welfare are shifting, exemplified by free contact systems being replaced by protected contact and use of target training (Clubb & Mason, 2002). Workshops focusing on elephant welfare and positive training methods have been conducted for mahouts in Myanmar, so the new generation of mahouts may be more conscious of welfare issues, though we did not see any influence of mahout age.

Animal behaviour is also influenced by HARs, and a range of behavioural tests have been used to assess this in livestock and zoo animals, often relying on testing fear of humans, such as through avoidance or vigilance tests (Carlstead, 2009). However, it is also valuable to monitor cooperative behaviours and animals’ affinity with caretakers, particularly in populations where there are a lot of interactions, such as with draught animals (Claxton, 2011). Our behavioural tests measured both affinity with specific mahouts (familiar mahouts) as well as general fear of humans (unfamiliar mahouts) and found elephants performed better at tasks with familiar mahouts and responded faster to those they had known for longer, suggesting specific relationships are important and distinct from general fear of humans. Over a third of elephants responded only to their own mahout, consistent with past interviews of mahouts in Myanmar and Nepal (Crawley et al., 2019; Hart, 1994). Older elephants responded more to their own mahouts, successfully completing the task >99% of the time, whereas younger elephants may be less used to responding to commands, irrespective of mahout identity. Elephant success at the task was less dependent on relationship length in males than females and in general, males were more successful than females particularly those with shorter relationship lengths. Males may be more accustomed to adjusting and responding to different mahouts as they are regarded as more difficult to manage and therefore change mahouts more often; in our study the average mahout relationship was 1.86 years for females compared to 1.44 years for males (Fig. S1). Elephants’ higher success and faster responses to more familiar mahouts suggest they were more active and alert when responding to their own mahout, and therefore disrupted relationships may reduce working efficiency. Mahouts have previously reported that elephants act slowly or even dangerously with other mahouts and that it takes ~3 years to develop an understanding and 5 years to build trust with an elephant (Hart, 1994; Mumby, 2019; Srinivasaiah et al., 2014). Interestingly, command rate was linked to elephants’ initial responses to commands and not success, suggesting it is important to get an elephant’s attention, but not necessarily to communicate beyond that. Although outside the scope of this study, future behavioural experiments could benefit from measuring other variables related to command style, such as both verbal (e.g. pitch, intensity), and non-verbal (e.g. position, distance) qualities, found to be important in human–dog communication (Fukuzawa et al., 2005; Gibson et al., 2014). A study on zoo animal responses to keepers found both non-verbal and verbal cues were important, but specific relationship measures were not assessed (Carlstead, 2009). Relationship quality could have serious repercussions for mahout safety; trends suggest zoo animals may be more likely to attack when cared for by many keepers, or by new, unfamiliar keepers (Hosey & Melfi, 2014a), an issue that could unfortunately be relevant to this and other captive elephant populations, with past estimates of 10–20 mahout fatalities occurring annually in MTE (Lair, 1997).

To conclude, there was no evidence that physiological stress responses were affected by mahout relationship measures in this study, although this may be because change is the new norm. In fact, longer mahout overall experience was linked to markers of increased elephant muscle damage and to some extent inflammation, perhaps due to harder work tasks or general complacency over time. By contrast, markers of muscle damage were reduced with longer specific relationships, likely as building trust and understanding reduces the need to use force to control elephants.Elephants require a behavioural adjustment period after changing mahouts as they take longer to understand less familiar mahouts, with important implications for the growing number of elephants used in tourism, whose behaviour must be controlled in unpredictable environments for the safety of handlers and visitors alike. As wild populations decrease, *in situ* conservation is becoming vital, and we must understand the effects of HARs on animals’ well-being to improve both welfare and conservation outcomes. Here we provide key information of how specific relationships influence the physiology and behaviour of an endangered species with around a quarter of their population in captivity. Still, further research is needed to understand how the underlying management system contributes to welfare effects (e.g. in free contact vs protected contact/*in situ* vs *ex situ* environments), and how impacts vary among species (social vs solitary/draught vs zoo management), to understand the true cost of human management to animal welfare and handler safety, applicable to billions of captive animals around the world.

## Funding

This work was supported by The European Research Council, The Academy of Finland and The Kone Foundation.

## Credit author statement

J.C.: conceptualization, methodology, investigation, formal analysis, data curation, writing—original draft, visualization; O.L.: methodology, formal analysis, data curation, visualization; D.F.D.S.: methodology, data curation; J.B.: writing—review and editing, validation, methodology; U.K.N.: investigation, resources, project administration; H.H.A.: investigation, resources, project administration; W.H.: resources, investigation, project administration, supervision; Z.M.O.: resources, project administration, supervision; M.S.: methodology, investigation; J.W.: investigation, data curation; M.L.: conceptualization, methodology, writing—review and editing, funding acquisition, supervision; V.L.: conceptualization, methodology, writing—review and editing, funding acquisition, supervision.The authors declare no conflict of interest in this study.

## Supplementary Material

Appendix_S1_coaa16
