# Supplementary material for: Influence of handler relationships and experience on health parameters, glucocorticoid responses and behaviour of semi-captive Asian elephants
Source: Conserv Physiol. 2021 Jan 6;9(1):coaa116. doi: 10.1093/conphys/coaa116 (PMC8528106; doi:10.1093/conphys/coaa116)
Supplement: Appendix_S1_coaa16 [file Appendix_S1_coaa116.docx]

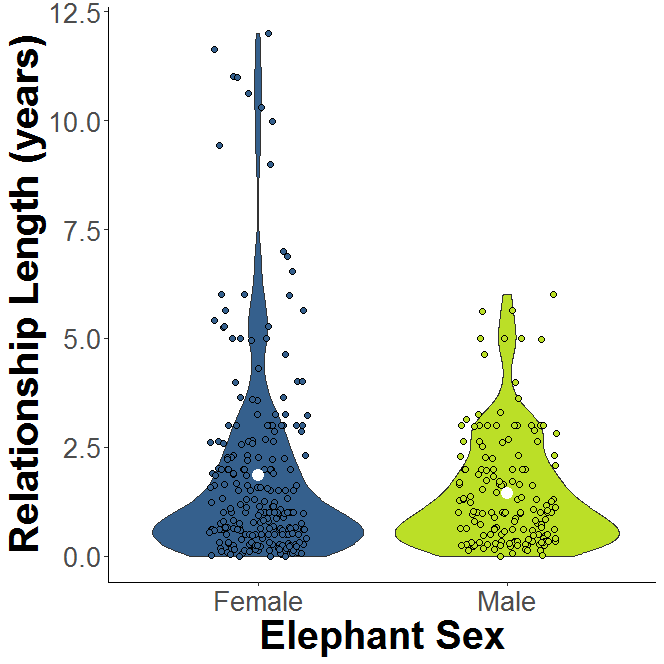


**Figure S1. Relationship length between elephant and mahout depending on the elephant’s sex.** White point shows the mean and coloured points the raw data.

**Tables S1i & S1ii. Outputs from *lmer* models assessing how elephants’ faecal glucocorticoid metabolite concentrations relate to i) their relationship length with their mahout and ii) their mahout’s total experience and age.** Reference season is cold and reference sex is female. Effects where p<0.05 are shown in bold

**Tables S2i & S2ii. Outputs from *lmer* models assessing how elephants’ H:L ratios depend on i) their relationship length with their mahout and ii) their mahout’s total experience and age. Reference season is cold and reference sex is female.** Effects where p<0.05 are shown in bold.

**Tables S3i & S3ii. Outputs from *glmmTMB* models assessing CK values of elephants depending on i) their relationship length with their mahout and ii) their mahout’s total experience and age. Reference season is cold and reference sex is female.** Effects where p<0.05 are shown in bold.

**Tables S4i & S4ii. Outputs from *lmer* models of elephants’ TWBC counts depending on i) their relationship length with their mahout and ii) their mahout’s total experience and age. Outputs for ii) are given both for a model with and without the term mahout age. Reference season is cold and reference sex is female.** Effects where p<0.05 are shown in bold.

**Tables S5i & S5ii: Outputs from *brm* models assessing how elephants’ successes at behavioural tasks depend on i) the calling mahout’s identity and ii) their relationship with the calling mahout.** Reference mahout identity is other and sex is female. Effects where Credible Intervals do not encompass zero are shown in bold.

**Tables S6i & S6ii: Outputs from *glmmTMB* models assessing elephants’ response times depending on i) the calling mahout’s identity and ii) their relationship length with the calling mahout. Reference mahout identity is other, sex is female.** Effects where p<0.05 are shown in bold.
